# Thin Film Encapsulation for LCP-Based Flexible Bioelectronic Implants: Comparison of Different Coating Materials Using Test Methodologies for Life-Time Estimation

**DOI:** 10.3390/mi13040544

**Published:** 2022-03-30

**Authors:** Anna Pak, Kambiz Nanbakhsh, Ole Hölck, Riina Ritasalo, Maria Sousa, Matthias Van Gompel, Barbara Pahl, Joshua Wilson, Christine Kallmayer, Vasiliki Giagka

**Affiliations:** 1 Department of Microelectronics, Delft University of Technology, 2628 CD Delft, The Netherlands; k.nanbakhsh@tudelft.nl (K.N.); v.giagka@tudelft.nl (V.G.); 2 Department of System Integration and Interconnection Technologies, Fraunhofer Institute for Reliability and Micro-Intregration IZM, 13355 Berlin, Germany; ole.hoelck@izm.fraunhofer.de (O.H.); barbara.pahl@izm.fraunhofer.de (B.P.); joshua.wilson@izm.fraunhofer.de (J.W.); christine.kallmayer@izm.fraunhofer.de (C.K.); 3 Picosun Oy, Tietotie 3, 02150 Espoo, Finland; riina.ritasalo@picosun.com; 4 CorTec GmbH, 79108 Freiburg, Germany; maria.sousa@cortec-neuro.com; 5 Comelec SA, 2301 La Chaux-de-Fonds, Switzerland; m.vangompel@comelec.ch

**Keywords:** flexible bioelectronics, thin-film encapsulation (TFE), liquid crystal polymer (LCP), atomic layer deposition (ALD), Parylene-C (ParC), long-term encapsulation

## Abstract

Liquid crystal polymer (LCP) has gained wide interest in the electronics industry largely due to its flexibility, stable insulation and dielectric properties and chip integration capabilities. Recently, LCP has also been investigated as a biocompatible substrate for the fabrication of multielectrode arrays. Realizing a fully implantable LCP-based bioelectronic device, however, still necessitates a low form factor packaging solution to protect the electronics in the body. In this work, we investigate two promising encapsulation coatings based on thin-film technology as the main packaging for LCP-based electronics. Specifically, a HfO_2_–based nanolaminate ceramic (TFE1) deposited via atomic layer deposition (ALD), and a hybrid Parylene C-ALD multilayer stack (TFE2), both with a silicone finish, were investigated and compared to a reference LCP coating. T-peel, water-vapour transmission rate (WVTR) and long-term electrochemical impedance spectrometry (EIS) tests were performed to evaluate adhesion, barrier properties and overall encapsulation performance of the coatings. Both TFE materials showed stable impedance characteristics while submerged in 60 °C saline, with TFE1-silicone lasting more than 16 months under a continuous 14V DC bias (experiment is ongoing). The results presented in this work show that WVTR is not the main factor in determining lifetime, but the adhesion of the coating to the substrate materials plays a key role in maintaining a stable interface and thus longer lifetimes.

## 1. Introduction

Electrical stimulation and recording of active tissues in the human body via biomedical implants has enabled physicians to enhance the quality of life for people who suffer from different neurological diseases [1,2,3]. To function, such implants need to have electronic circuitry, which usually takes the form of silicon chips with integrated circuits, additional passive components and stimulation/recording electrodes [4]. By themselves, these electronic systems do not have sufficient protection to survive the aggressive humid environment of the body, while at the same time the body must be protected from them; therefore, they must be well packaged. Conventional methods of packaging electronic implants in titanium metal housing are always tested for their hermeticity using helium leak tests to ensure a long-lasting robust protection in the body [5,6]. These devices, however, are limited by their rigidity and large size. Such bulky packages do not allow the implant’s electronics to be near the delicate and soft tissue that is the target for stimulation and/or recording. To bridge this distance, the implant’s electronics are connected with special leads to the stimulated region. This creates many possible failure points, restricts mobility, occasionally results in infection and can cause discomfort for the patient [7,8]. Therefore, there is an emerging need to miniaturize the packaging so that the electronics can be in closer proximity to the target tissue. This is of particular importance in the field of Bioelectronic Medicine, where it is envisioned that therapy in the form of electrical impulses will be delivered through not one, but a network of these devices, distributed in the body, operating in synchrony [4]. 

**Figure 1:**
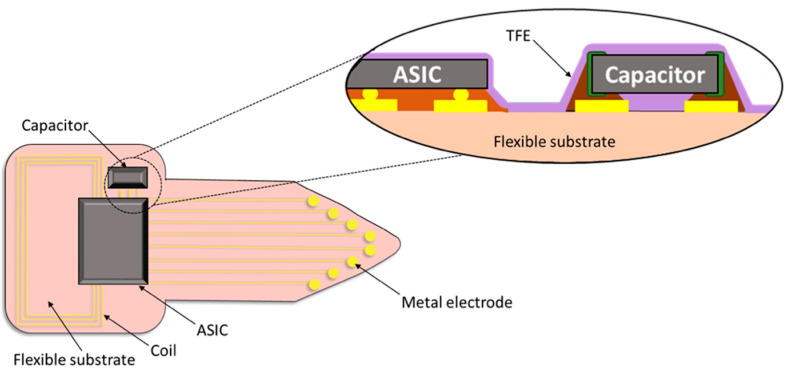
Generic sketch of a wirelessly powered fully implantable active device based on a polymer substrate: electronic components in the form of capacitor and ASIC constituting the functional circuit; metal electrodes for delivering or receiving signals to/from the tissue; coil for wireless communication and power transmission. All implemented into a flexible biocompatible polymer covered with a thin film (TFE) encapsulation layer.

Flexible substrates, such as polyimide (PI), Parylene C (ParC), polyurethane (PU), liquid crystal polymer (LCP), and silicone elastomers have been used in the electronics and wearable devices industries as substrates for electronic components and metal interconnects. Although, in the past decades these polymers also gained increased attention for realizing flexible neural interfaces in the form of micro electrode arrays (MEAs), most reported MEAs are tethered to an external unit that holds the electronics used for stimulation and/or recording [9,10,11,12]. This often renders the device suitable only for short-term studies. Moving towards fully implantable, biocompatible, yet flexible, active implants (generic realization example shown in Figure 1) requires a tailored packaging approach.

LCP has gained attention as a new substrate material for implants due to the following advantages: low moisture permeability [13], thermoplasticity, high temperature compatibility and mechanical stability. Monolithically fabricated LCP-based passive neural electrode arrays have been reported with lifetimes longer than 300 days at 75 °C [14], and up to 158 days at 87 °C [15], in phosphate-buffered saline (PBS) solution. This process is based on the fusion of LCP layers during lamination, preventing water ingress and water vapor condensation in the LCP-LCP interface, which is a common cause of delamination and further failure for polymer-based and encapsulated devices [16,17,18,19]. The LCP-based fabrication process can be used for creating both passive and active neuroprosthetic devices. Thermal lamination enables monolithic encapsulation of the embedded metallization [10,15], while injection molding [20,21], jet deposited glob-top [22], multilayer stacking [23] or thermoforming of LCP-lids [14] can be used for encasing the electronic components. 

ALD is a chemical vapor deposition technique that has been widely investigated in the last decade for coating medical devices [24,25,26]. Particularly, Al_2_O_3_, HfO_2_, and TiO_2_ oxide layers demonstrated low moisture permeability and conformal coverage with a low density of pinholes [27]. Al_2_O_3_-based ALD also exhibited excellent thermal and mechanical properties, while HfO_2_-based ALD has high chemical stability in ionic media and anti-corrosion properties [28,29]. In fact, HfO_2_-based ALD encapsulation layers with silicone finish have exhibited a long lifetime of over 1028 days at 60 °C in PBS in accelerated aging tests [30]. Using ALD layers in combination with organic Parylene C layers, which offers conformal coating of sharp edges and gaps down to microns size and is also able to serve as a buffer for the high-barrier inorganic ALD layers, could possibly prolong the lifetime of the implant. This is due to an expected increased time needed for molecules to travel through such a multilayer coating to reach the electronics [31,32]. 

In this paper, to the best of our knowledge, we investigate for the first time the feasibility of using TFE materials with a silicone elastomer finish as a packaging solution for LCP-based implantable bioelectronics. Toward this goal, and as a first step, pre-screening tests were carried out to evaluate the interface adhesion of the TFE materials to LCP sheets. This process was used to optimize the deposition parameters of the encapsulation material on LCP substrates. Thin coatings were deposited on LCP substrates and soaked at elevated temperature in saline and analyzed. LCP-on-LCP structures were also fabricated and used as a reference for these investigations. To further evaluate the adhesion of the TFE coatings, T-peel tests were performed. Although, strong adhesion between the layers can be one of the indicators for long-term performance of the implants, water tightness will also protect the implant from moisture. Therefore, water-vapor transmission rates (WVTR) were calculated for the same coatings. Bending of the coated LCP substrates was performed to take into account the fragility of the thin films, when applied to a flexible substrate. Finally, sensitive impedance measurements were performed on interdigitated comb (IDC) metal structures during a long-term accelerated aging study to estimate the lifetime of the selected coating on LCP. Results show that thin-film encapsulation with a silicone finish can indeed be a viable solution for packaging LCP-based bioelectronics, achieving lifetimes comparable to other LCP-based packaging techniques.

## 2. Materials and Methods

### 2.1. Preparation of Thin Film Encapsulation (TFE) Layers

For this work a number of test structures on LCP substrates have been prepared and evaluated using two different TFE variations, as described below.

For type 1, a 100 nm HfO_2_-based ALD stack (referred to in this paper as ALD ML or TFE1), consisting of multiple HfO_2_-based ALD layers was deposited at Picosun Oy using the Picosun^®^ R-200 Advanced ALD reactor, at a pressure of about 1 mbar (N_2_ atm.). The PicoHot source system (PH-300) and PicoSolution (both Picosun Oy) precursors were vaporized from stainless-steel precursor bottles at increased and room temperature, respectively. Thermal ALD-processes at 125 °C were applied with a build up the HfO_2_-based coating. For the ALD deposition, the LCP samples were placed on top of a Si pocket wafer since only a top coating was needed.

For type 2, a 7 µm stack consisting of Parylene C and Al_2_O_3_ and TiO_2_ ALD multilayers (referred to as ParC hybrid or TFE2), were deposited at Comelec using the C30H Parylene-ALD hybrid deposition system. More specifically, each TFE2 consists of 1–3 µm Parylene C/3x [Al_2_O_3_/TiO_2_/Parylene C]/4–6 µm Parylene C. Both ALD and Parylene C depositions are performed consecutively in the same vacuum vessel and without venting cycles. This eliminates any contamination between the polymer and metal-oxide layers usually present after handling substrates. The Parylene C layers were deposited at room temperature. The system includes an O_2_ plasma treatment step followed by a silane adhesion promoter processing step. The deposition of the oxide layers is performed at temperatures below 100 °C. The same as for TFE1, precursors were vaporized from stainless steel bottles at increased or room temperature. A more detailed description of the process is explained in [31].

**Figure 2:**
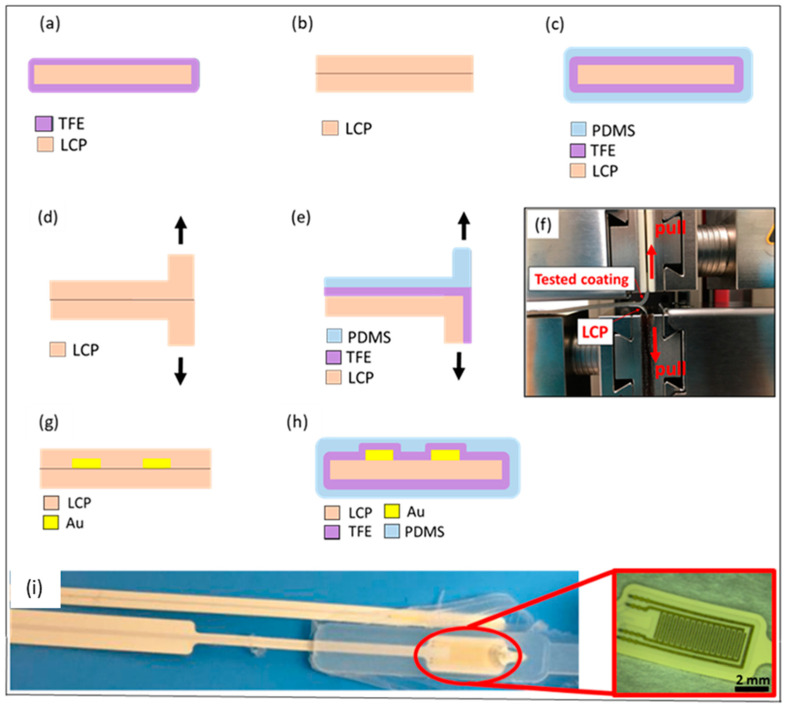
Schematic cross-section illustration of the tested samples: LCP-TFE1/2 samples used for pre-screening test (**a**), LCP-LCP laminate (**b**) and LCP-TFE1/2 with silicone finish samples (**c**) used for sorption tests, LCP-LCP (**d**) and LCP-TFE1/2 (**e**) with silicone finish samples used for adhesion T-peel test. Adapted ASTM D1876 T-Peel test setup with the sample (**f**). Schematic representation of the IDC tested samples: LCP-Au-LCP (**g**) and LCP-Au-TFE1/2 with silicone finish (**h**). Exposed Au IDC test structure on LCP (**i**).

For the silicone elastomer finish, a polydimethylsiloxane (PDMS) layer (MED2-4213, NuSil Carpinteria, Carpinteria, CA, USA) was deposited on top of TFE1 and TFE2. For silicone deposition, vacuum centrifuging followed by curing under high pressure was used to ensure conformal void-free coating. This additional silicone layer has well documented biocompatibility and its Young’s modulus is closer to that of soft tissue, hence it is the material of choice to directly interface with tissue. More importantly for this application, as we have previously shown that, when used together with thin plasma enhanced chemical vapor deposition (PECVD) or ALD films, silicone finish coating can offer additional protection to the active device by filling any defects or pores of the ceramic, thus extending the expected lifetime [30,33]. TFE1-silicone and TFE2-silicone coated test structures were tailored to T-peel tests, WVTR evaluation and impedance spectrometry. As a reference, LCP-on-LCP (LCP laminate) samples were also fabricated and included in the investigations. All the materials configurations used for different tests are schematically presented in Figure 2 and explained in detail in the following sections.

### 2.2. Evaluation Procedures

#### 2.2.1. Pre-Screening of the Coatings

For the pre-screening test TFE1 and TFE2 were deposited on LCP substrates as shown schematically in Figure 2a. Soaking of the samples was performed in a water tank that was placed on a hot plate with a thermocouple. The temperature was set at 67 °C and 65 °C for TFE1 and TFE2, respectively. The water tank was sealed with parafilm and aluminium foil to limit evaporation. The samples were placed in glass vials with a top cover. Each sample was placed in a separate vial filled with PBS. Salinity and pH were checked regularly. Pre-screening was based on optical inspection and cross-section microscopy analyses of the TFE coatings. Scanning electron microscopy (SEM) images of bare LCP and TFE1-covered LCP samples were taken to validate the coating’s conformability. For a more detailed analysis, cross-sectioning of the LCP-ALD interface was performed on a FEI Tecnai Osiris (Scanning) Transmission Electron Microscope S/TEM, using the in situ-focused ion beam (FIB) lift out technique. Cross-sections of LCP-ALD interfaces were investigated before and after a 2-month aging study at 67 °C PBS. This was carried out to evaluate the stability of the coating together with its adhesion to the LCP substrate in an environment mimicking the physiological fluids in the body.

#### 2.2.2. Sorption Tests and WVTR Calculation

WVTRs were calculated from sorption curves analysis obtained using a sorption analyser (SA Q5000, TA Instruments, New Castle, DE, USA). The procedure was as follows: samples (Figure 2b,c) of approximately 5 × 5 mm² were cut and dried at 80 °C for at least 3 h; the samples were mounted onto a quartz pan and exposed to 60 °C/60% relative humidity (RH) atmosphere; the weight gain was monitored over a period from 24 to 36 h, until saturation was reached. WVTR values were then estimated using the ratio between the permeance Π and the partial pressure of the water vapor Δ*p* (Equation (1)):

*WVTR* = Π·Δ*p* (1)

where permeance of a film with a membrane thickness *d* and permeability *P* is Π *= P/d.* Introducing the solubility *S*, as the relation between *C_sat_*—saturation concentration and Δ*p* (*S = C_sat_*/Δ*p*), and diffusion coefficient *D*,

*P = S·D = C_sat_/*Δ*p·D* (2)

Substituting Equation (2) into Equation (1) gives Equation (3) for calculation of the water vapour transmission rate:

*WVTR = C_sat_·D/d* (3)

This relation contains some simplifications (e.g., high upstream vapor pressure and a near zero downstream vapor pressure), which is why it is used here for comparison rather than for calculation of absolute values.

Samples in the bended state were prepared by constraining the samples into a curved shape using a small pan, achieving a radius of curvature of about 5 mm, which was upheld during the whole measurement period of 24 to 36 h.

#### 2.2.3. Adhesion Evaluation by Adapted ASTM D1876 T-Peel Test

Adhesion testing was performed on the LCP laminate sample (Figure 2d) and the LCP-TFE1-silicone and LCP-TFE2-silicone stacks (Figure 2e). An adapted ASTM D1876 T-Peel test was performed as follows: a strip of one of the two bonded materials with a small unbonded area for clamping is prepared; the top and bottom are pulled apart at a constant speed (100 mm/min); the force required to peel the two materials is recorded; the test ends when the sample is completely peeled or one of the materials ruptures (Figure 2f). The samples were tested before and after soaking for 24 h at 60 °C in 0.9% saline solution. SEM and energy-dispersive X-ray spectroscopy (EDX) analyses (Zeiss Supra 55 VP, 25 kV, working distance of 8.5 mm) of the LCP-TFE2 and PDMS surfaces were performed for T-Peel tested samples before and after soaking to evaluate the adhesion of the coating. At least three samples were used for each of the tests and the average values are presented in the results.

#### 2.2.4. Electrochemical Impedance Spectrometry (EIS) on IDC Structures

The long-term performance of the thin encapsulation layers was evaluated by EIS measurements of gold (Au) IDC structures. 3 µm thick Au IDCs were electroplated on top of a structured palladium (Pd) seed layer deposited on LCP. For the IDC, 30 fingers were used with a gap of 100 µm, where the width of the Au metallization was also 100 µm. The IDC dimension were designed to be 2 mm by 5 mm. A more detailed fabrication procedure of Au IDCs on LCP has been provided in [10]. After the thin coating deposition, the silicone finish was applied. More specifically, samples were further covered with a 0.5 mm thick low viscosity silicone layer from both sides to fill in the possible defects or pores in the thin encapsulation layer. The IDC test structure is schematically shown in Figure 2g,h. For EIS, all samples were connected to a Solatron Analytic Modulab XM^®^ potentiostat and the impedance between the two metal combs was measured with the samples being immersed in PBS solution at 60 °C. The recorded spectrum for all the EIS measurements was between 0.1 Hz and 100 KHz.

For a bioelectronic implant, besides moisture, the materials and interfaces will also be subjected to electric fields generated by the voltages on the metal traces. Such electric fields could lead to earlier failure of the device. Given that DC signals have been reported to accelerate failure [34], therefore a group of samples was exposed to a continuous 14 V DC signal. For long-term EIS characterization and accelerated aging, a dedicated set-up was realized according to [35]. Biweekly in situ impedance measurements were recorded to monitor the performance of the TFE-silicone coatings over time. A sample was considered to have failed once the impedance magnitude at 0.1 Hz had deviated more than 10% from the original values measured at the beginning of the aging study.

## 3. Results

### 3.1. Pre-Screening of the Coatings by Soaking and Optical Inspection

The coatings were initially evaluated for stability and absence of visible delamination from LCP, immediately after deposition and then after soaking at accelerated temperatures for 2 months. The LCP coating on the LCP substrate was eliminated from the pre-screening tests, due to pre-existing evidence of excellent performance [14,15].

**Figure 3:**
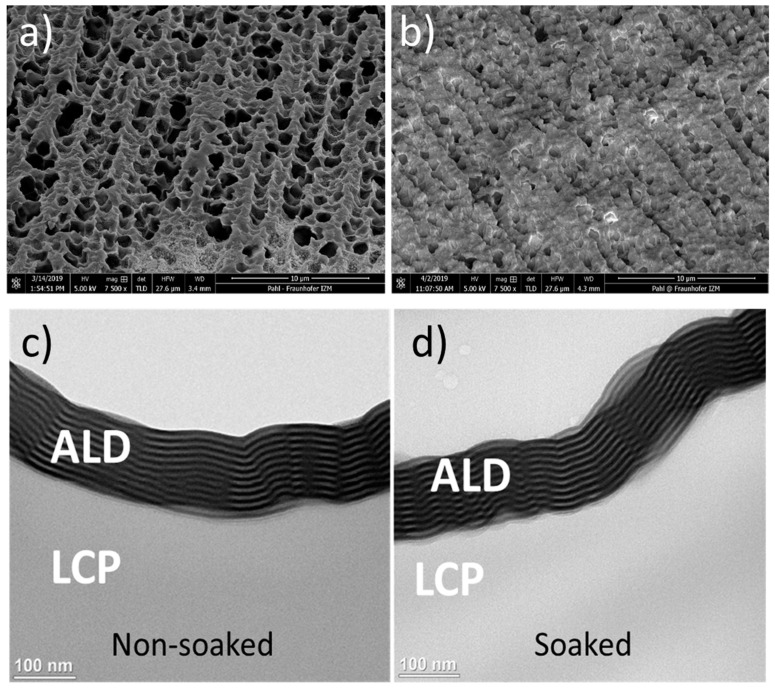
SEM images of bare LCP (**a**) and LCP coated with a 100 nm thick HfO_2_-based ALD ML (TFE1) (**b**). Cross-sectional TEM images of 100 nm HfO_2_-based ALD ML on LCP: (**c**) before soak; (**d**) after 2 months soak in PBS at 67 °C.

TFE1 was deposited on a rough LCP substrate; SEM images before and after TFE1 deposition are shown in Figure 3a,b. After deposition the sample was soaked for 2 months at 67 °C. TEM images of the cross-section of the nanolaminate ALD layers are presented in Figure 3c,d. The HfO_2_-based ALD ML coating conformally covers the rough topography of the LCP substrate (Figure 3c) and stayed intact after the soak (Figure 3d).

Similarly, a coating based on TFE2 was deposited on rough LCP substrates; different parameters, such as ALD thicknesses, pre-treatment, adhesion promoters, number of dyads, ParC interlayer thicknesses were varied; the samples were soaked for 2 months at 67 °C; if no visible signs of delamination were observed the process was selected for further tests.

### 3.2. Barrier Properties Evaluation with Sorption Test

Sorption tests were used to calculate the water vapor transmission rate of the coating materials. A low WVTR indicates better moisture protection. As shown in Table 1, WVTR can be reduced by approximately one hundred times by the addition of TFE1-silicone or TFE2-silicone coatings to bare LCP. The WVTR of the TFE1-silicone increased after bending due to the fragility of the ceramic layers, however it remained lower than for bare LCP. Bare LCP was assumed to have the same WVTR before and after bending. TFE2-silicone demonstrated better performance than TFE1-silicone after the bending.

The values obtained for the WVTR and T-Peel tests are given to compare the coatings with each other, rather than being absolute values. This is due to the assumptions mentioned in Section 2.2.2, as well as the fact that the WVTR values were derived from the Formulas (1)–(3) using the values obtained during the sorption test and not measured directly as during the standard MOCON test. Nevertheless, the values obtained for pure LCP were still comparable to the values in the literature [36], despite the slightly different setup conditions (60 °C/60% relative humidity, instead of 37 °C/100% relative humidity).

### 3.3. Adhesion Evaluation with T-Peel Test

Table 2 gives the average force needed to compromise the adhesion inside each material stack under test, both prior to, and after soaking.

**Figure 4:**
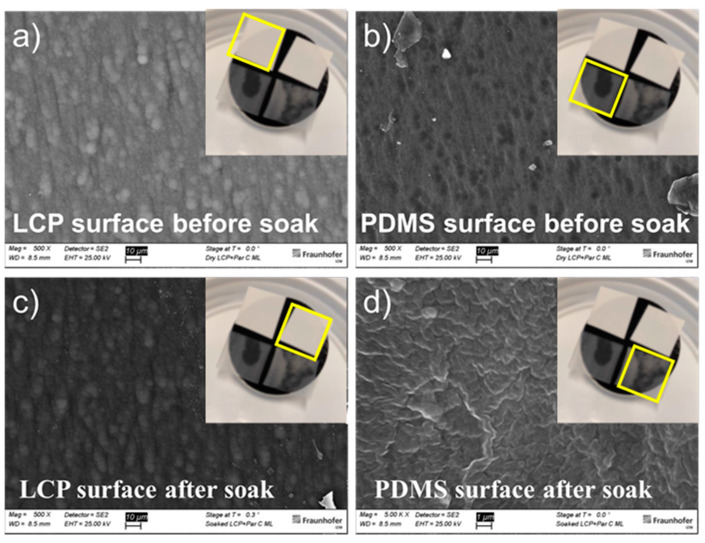
SEM images of LCP (**a**,**c**) and PDMS (**b**,**d**) surfaces after T-peel test before and after soaking.

Soaking for one day did not have an effect in the adhesion properties of the investigated stacks. The LCP-on-LCP reference samples could not be peeled apart. For the LCP-TFE1-silicone a force of 8 N was required to delaminate TFE1 from the LCP. The integrity of the LCP-TFE2-silicone stack was compromised much earlier, at 0.1 N, however, at a different interface, between TFE2 and silicone. The force required to peel TFE2 from LCP was therefore not possible to record. In fact, Parylene C, which is the topmost layer of TFE2, has poor adhesion to silicone and this was proven by SEM-EDX spectroscopy analysis presented in Table 3 and Figure 4, which revealed the presence of TFE2 in the form of chlorine, aluminium and titanium on LCP after the T-peel test, for both, soaked and non-soaked samples.

### 3.4. Coating Performance Evaluation with IDC Structures

**Figure 5:**
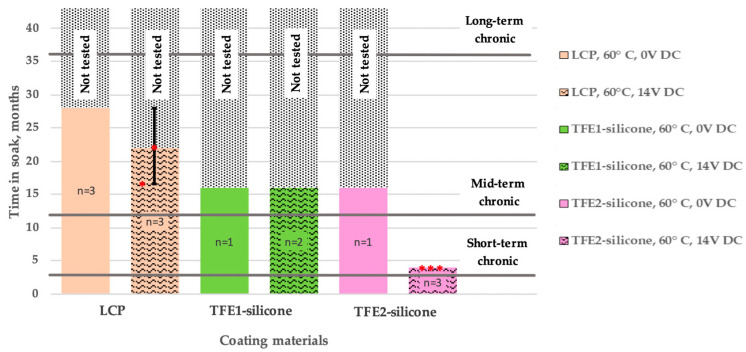
Lifetime of different coatings on LCP substrates with Au IDCs in 60 °C PBS. End of a sample’s lifetime was indicated by a >10% deviation of the impedance magnitude at 0.1 Hz, compared to the original value at the beginning of the aging study. Red asterisks (*) represent failed samples at the corresponding month.

Comb metal structures are highly sensitive in detecting the slight changes related to the water vapour condensation between the metalization or any change in the dielectric properties of the insulating materials [37,38]. Figure 5 provides the lifetimes of all the samples in the long-term aging study, recorded until the submission date of this manuscript. Note that the non-failed samples from all three categories are still in test. The LCP-LCP samples were the first group of samples placed in the accelerated aging study, later followed by the thin-film encapsulated samples, thus has so far been tested for longer. The provided lifetimes are those have so far been achieved for each coating. Only in one case, specifically for the TFE2-silicone coatings under 14 V bias, the experiment has stopped as all samples reached the end of the lifetime. 

The LCP-LCP samples all showed stable impedance spectra throughout the first year of the aging study. Extending the study duration, however, led to two of the biased samples failing at months 15 and 22, respectively. Failure analysis using X-ray microscopy showed a crack/corrosion in a metal trace leading to the IDC, possibly due to impurities in the gold metallization. Light microscopy did not reveal any signs of corrosion in the interdigitated area suggesting a hydrolytically stable LCP-LCP lamination between the comb structures. Thin-film-encapsulated IDC structures were in test for 16 months (until the date of submission). For the TFE1-silicone coated IDCs, both non-biased and biased samples showed stable impedances throughout the 16-month duration of soaking. The TFE2-silicone coated IDCs showed failure only when subjected to the 14 V DC bias signal. Failure occurred around month 4 of the study when significant impedance drop was recorded, suggesting a gross leakage path in between the comb structures. 

## 4. Discussion

### 4.1. Methods Used for the Evaluation of the Proposed TFE Coatings

Since there is no established method for the evaluation of the lifetime of TFE and polymer coatings for active neural implants, the presented values and methodologies have limitations that must be considered. These are summarized below.

Predicted lifetimes are commonly, in the literature, calculated from empirical Arrhenius modelling of accelerated reactions, with an acceleration factor Q_10_ = 2, which states that a rise in temperature of 10 °C will cause approximately a doubling of the rate of chemical reaction [39]. This calculation has its limitations since it does not consider activation energies for the given processes due to practical reasons, and can therefore lead to over or under-estimation of expected lifetimes. Furthermore, it is likely that an increased temperature could cause new failure mechanisms in polymers which would not happen at 37 °C. Due to the above, we have selected to employ a conservative approach and abstain from translating our results to expected lifetimes at body temperature.

The PBS solution used for soaking the IDC structures cannot fully mimic the aggressive physiological body environment in terms of the expected hydrolytic, oxidative, and enzymatic reactions. This limitation of aging in PBS is well known and attempts to overcome it, by using hydrogen peroxide instead, have appeared in the literature. The use of hydrogen peroxide as a soak medium aims to mimic the presence of reactive oxygen species comparable with immune system attack [40].

Since due to the flexibility and softness of the LCP substrate it was not possible to use the ASTM D3359 tape test, which is commonly used for adhesion evaluation of the coatings and classifies the obtained values into categories, an adapted ASTM D1876 T-Peel test was used for determining the relative peel resistance. According to the conventional ASTM D3359, a coating which remains intact after applying a 7 N force is classified as 5B and indicates strong adhesion. If we compare it to the relative peel resistance of 8 N, obtained during the LCP-TFE1 T-peel test, it would also correspond to strong adhesion. Nevertheless, we have used the results obtained from our test to estimate relative (among compared coatings) performance of the adhesion to the substrate, and forces reported here are not equivalent to the ASTM D3359 classification.

Similarly, the values obtained for the WVTR are also used to compare the coatings to each other, rather than being absolute values. This is due to assumptions and adoption of the tests to our coatings discussed in Section 2.2.2 and Section 3.2.

### 4.2. Performance of LCP and TFE Coatings

Considering the results presented in this paper, each of the coating options comes with its own advantages and shortcomings. Below we summarize further findings regarding the chosen encapsulation materials that were drawn from our experiments.

*LCP-on-LCP*

Despite having lower barrier properties to moisture ingress, in comparison with the other two coatings as shown in Table 1 and reported in previous literature [13], LCP encapsulated IDC structures can remain functional for up to 28 months at 60 °C in PBS solution with a 14 V continuous DC bias. Although the acceleration factor from the constant 14 V DC bias voltage cannot be easily derived, the long-lasting performance of the IDC impedance shows that the dielectric properties of the LCP material are outstanding. The stable dielectric properties can be attributed to the low water uptake of LCP together with its high hydrothermal stability. Between the metal combs, the fusion of the two LCP films prevented any moisture appearance that could otherwise create a shunt leakage path, altering both the impedance magnitude and phase profiles of the EIS measurements. However, the limitation of the LCP-LCP lamination is that it cannot cover 3D components, such as discrete passive components, without additional processing steps, since the lamination technology will either destroy the components, or it will not allow for a conformal coverage of thick components with complete merging of LCP layers. Therefore, LCP-LCP lamination can only be the optimal encapsulation solution for metal traces, but not for discrete electronic components. For such systems, the other two TFE strategies mentioned below are recommended.

*LCP-TFE1-silicone*

In comparison with the bare LCP, the ALD nanolaminates of TFE1 exhibited better barrier properties, see Table 1 with calculated WVTR values comparable with those available in the literature [41]. Further advantages of this method of encapsulation include conformal cover of the substrate, shown by TEM imaging (Figure 3c,d) and improved adhesion to LCP, shown by T-peel test (Table 2). However, the very thin ALD layers form a fragile stack, and bending of the samples significantly increased the WVTR of the TFE1-silicone coated sample from 2.87 to 68.61 mg/m^2^ per day. This puts a significant barrier for the adoption of an encapsulation stack based on ALD with silicone finish on flexible/bendable substrates. Nevertheless, IDC samples coated with TFE1-silicone maintained their functionality for at least 16 months with the 14 V DC bias (no bending, experiment ongoing).

*LCP* *-TFE2-silicone*

The investigated TFE2–silicone exhibited the best barrier properties, which are very close to the ones exhibited by TFE1–silicone, but crucially the integrity of the moisture barrier was not as significantly compromised by bending (Table 1). This can be explained by the fact that the Parylene C layers, which were part of the TFE2 stack, keep the intermediate thin ceramic layers together due to a strong interlayer adhesion and prevented the fast water ingress, even after bending, by creating a tortuous pathway along the deposited layers, which has been more explained in detail in [31]. A higher moisture barrier will delay polar water molecules reaching the critical LCP-Parylene C interface. Adhesion within the LCP-TFE2–silicone stack, however, was lower compared to other investigated stacks, with the adhesion of the silicone to Parylene C being the lowest. This was proven by EDX analysis results (Table 3). Despite the low adhesion, based on the IDC results, the predicted lifetime of the TFE2-silicone coated samples is at least 16 months years at 60 °C (experiment ongoing), and 4 months when continuously applied to a 14 V DC bias. For longer-term applications, the weak adhesion observed in this work will be problematic; local delamination is expected, which would introduce stresses in the coating stack and, as a result, reduce the barrier properties. Thus, further improvement at the interface between Parylene C and silicone is needed. This could be achieved by the addition of PECVD ceramic interlayers [42,43]. Alternatively, TFE2 could be modified to finish at one of the Al_2_O_3_ and TiO_2_ ALD layers instead of Parylene C.

### 4.3. General Remarks

Figure 5 indicates that, even if projected lifetimes are estimated using a conservative approach (not taking into account any acceleration factor), all coatings considered here seem to offer promising encapsulation solutions at least for short-term (3 months) chronic applications, provided each coating is carefully chosen depending on the intended purpose. LCP coatings, as well as the ALD nanolaminates of TFE1 with a silicone finish, seem to be suitable also for mid-term chronic (~1 year) lifetimes, but TFE2-silicone should be optimized as suggested above. Longer chronic lifetimes (over 3 years) are significantly harder to achieve, and were not the focus of this work. In fact, looking at the performance of the LCP-on-LCP cohort which was tested for longer, we observe that the stability of the stack was compromised for longer test durations at least in some of the samples that were under 14 V DC bias.

As the results reported in this paper demonstrate the viability of using TFE for LCP based electronics, further evaluations are still needed to realize a fully implantable bioelectronic device based on LCP material. To begin with, optimization of the stacks and deposition conditions should be still performed. Longer lifetimes can probably be achieved if stronger interface adhesion is realized. For example, surface activation via oxygen plasma and reactive ion etching has been shown to create functional group on the LCP surface. Additionally, adhesion promoters could be investigated on the LCP substrate to create a long-lasting bond between the TFE and the LCP. More detailed chemical and structural analysis between LCP and thin coatings should be performed to study the interface and possible fusion into LCP. The encapsulation of any final assembled LCP substrates with 3D components (ASICs and passive components) is an ongoing investigation by the authors. The pilot tests on bended samples of coated LCP suggest that bending of the IDC samples should be performed to evaluate the behavior of the coating on metal structures. Cleaning and sterilization procedures during and after manufacturing steps should be implemented to the final process. Finally, in vivo characterization of the encapsulation performance should be performed and compared with the predicted lifetime of the coating calculated from the empirical Arrhenius modelling.

### 4.4. Choosing an Appropriate Coating for Each Application

Encapsulation of implantable electronics using polymers and thin film coatings is a promising solution to design miniaturized, soft, light-weight devices with high biocompatibility, transparency and flexibility. Although, all polymers are permeable to water vapor, if designed with the appropriately chosen materials and processing parameters they can provide implants with decades-long lifetimes [25,29,33]. Considering the results presented in this paper, each of the coating options comes with its own advantages and shortcomings. While choosing an appropriate coating for each application the following should be considered: (1) The adhesion of the encapsulation stack to the underlying layer should remain strong for the lifetime of the device. To the best of our knowledge, there is no minimal peel force value that is sufficient for long-term medical implant packaging. (2) Coatings that act as good barriers will not prevent, but only delay water/moisture ingress that would weaken the critical interfacial bonds. They are nevertheless beneficial to extend the time-to-failure. (3) The coating should conformally cover the underlying substrate to avoid creating any cavities and voids. (4) The coating together with the substrate material should both remain stable in wet ionic environments. Aging tests should be expanded to include applied bias and mechanical stress, both dependent on realistic estimates derived from the specific use case. In practice, conformal encapsulation stacks will rely on achieving a good balance among the aforementioned characteristics to achieve long implant lifetimes.

## 5. Conclusions

In recent years, numerous works have shown the potential of LCP in creating high resolution flexible MEAs [10,15,44,45]. With a view to realizing a fully implantable wireless device with on-board components (coils, interconnect metallization, ASICs, and passive components), TFE was proposed as a promising small form factor packaging solution.

ALD nanolaminate and ParyleneC-ALD hybrid multilayers have been investigated as two thin film encapsulation materials, strengthened by a silicone finish for LCP based electronic substrates. The encapsulation performance was evaluated using three different methodologies. More specifically the adhesion performance of the encapsulation layers on LCP foils was investigated before and after soaking at elevated temperatures, while the WVTR was calculated before and after bending of the samples. The lifetime performance of the two coating multilayers was evaluated using an accelerated aging study on IDC structures with a DC bias voltage. It was found that TFE-silicone can be a viable technique in packaging LCP based electronics for short to medium-term chronic applications, making this packaging solution a cheap and promising method in realizing fully implantable wireless MEAs.

## Figures and Tables

**Table 1:** WVTR of the coatings measured with a Sorption Analyser at 60 °C and 60% relative humidity.

Materials Stack	Thickness	WVTR	WVTR
			(After Bending ^1^)
LCP	100 µm	202.05 mg/m^2^ day	202.05 mg/m^2^ day
LCP–TFE1	100 + 0.1	2.87 mg/m^2^ day	68.61 mg/m^2^ day
LCP–TFE2	100 + 7	2.23 mg/m^2^ day	20.66 mg/m^2^ day

^1^ 5 × 5 mm^2^ samples were bent to a radius of curvature of about 5 mm for a period from 24 to 36 h (until saturation was reached).

**Table 2:** Peel force required to compromise the adhesion inside each material stack under test.

Materials Stack	Peel Force 1 (Before Soak)	Peel Force 2 (After Soak ^1^)
LCP-LCP	failed to peel	failed to peel
LCP-TFE1-silicone	8 N (btw. TFE1 and LCP)	8 N (btw. TFE1 and LCP)
LCP-TFE2-silicone	0.1 N (btw. TFE2 and silicone)	0.1 N (btw. TFE2 and silicone)

^1^ Samples were soaked for 24 h at 60 °C in 0.9% saline solution.

**Table 3:** Elemental analysis of the LCP and silicone (PDMS) surfaces after T-peel test using EDX.

	Before Soak		After Soak	
Element	On LCP	On PDMS	On LCP	On PDMS
C	80.41%	36.8%	80.13%	36.91%
O	0%	30.72%	0%	31.15%
Si	0.07%	32.48%	0.26%	31.94%
Cl	19.12%	0%	18.85%	0%
Al	0.23%	0%	0.34%	0%
Ti	0.17%	0%	0.11%	0%
